# An evaluation of the effects of universal free school meals on secondary school-aged pupils’ dietary intakes in England: a natural experiment

**DOI:** 10.1186/s12889-025-25960-7

**Published:** 2025-12-19

**Authors:** Suzanne Spence, John N. S. Matthews, Jayne V. Woodside, Robert Brownell, Katy Scammell, Jennifer Bradley

**Affiliations:** 1 Population Health Sciences Institute, Human Nutrition and Exercise Research Centre, Newcastle University, Framlington Place, Newcastle, NE2 4HH UK; 2 Biostatistics Research Group, Population Health Sciences Institute, Newcastle University, Newcastle, NE1 7RU UK; 3 Centre for Public Health, School of Medicine, Dentistry and Biomedical Sciences, Queen’s University Belfast, Institute of Clinical Science, Block A, Grosvenor Road, Belfast, BT12 6BJ UK; 4 London Borough of Tower Hamlets, London, E1 1BJ UK

**Keywords:** Schools, Universal school meals, Adolescents, Dietary intake, Nutrition

## Abstract

**Background:**

In September 2023, Tower Hamlets implemented a major policy change to Universal Free School Meals (UFSM) extending provision to all secondary school pupils aged 11-16y. There is limited evidence exploring the effects of UFSM in secondary school-aged children, and this, therefore, offered an opportune natural experiment to explore the effects of this policy change. The aims of the project were to explore the effects of UFSM on secondary-school aged pupils’ dietary intake and food security.

**Methods:**

Natural experiment using a mixed longitudinal cross-sectional design in intervention (*n* = 3) and control schools (*n* = 2). Schools purposefully selected Year 8 classes and all pupils were invited to participate using opt-out consent. Pupils completed four non-consecutive weekdays of total dietary intake using Intake24. Dietary outcomes were mean change in key macro- and micronutrients pre-post-UFSM at lunchtime and in total diet. Within-pupil changes were analysed using a linear model that adjusted for gender and included a lunch-type and intervention/control interaction.

**Results:**

Dietary data pre- and post- UFSM was captured for 176 pupils (99 intervention, 77 control). Only a small number of pupils in the intervention schools changed lunch-type to take up UFSM (*n* = 19). Most pupils had a combination of School, Home or Other lunch. Only two of the 18 tests for interaction gave *p* < 0.05, namely energy and protein intakes at lunchtime. Most pupils were food secure both pre- and post-UFSM in intervention and control schools.

**Conclusion:**

Although we found almost no lunch-type and intervention/control interaction effects on mean change in pupils’ dietary intakes, inferences about dietary effectiveness require careful consideration. Reasons include only a small number of pupils took up the UFSM offer, there may be a delay between implementation and take-up, and we do not know how the offer of UFSM was communicated to parents and/or pupils. This study highlights a need for better understanding of reasons for poor take up and whether take up will change over time post policy implementation. Wider challenges pertinent to school food, such as, the quality and availability of food on offer, and the dining environment, remain important considerations to maximise the nutritional benefit of universal free school meal policy changes.

**Supplementary Information:**

The online version contains supplementary material available at 10.1186/s12889-025-25960-7.

## Background

In England, since 2014, Universal Free School Meal provision (UFSM) enables all primary school children aged 4-7years attending a government funded school to receive a free school meal [1, 2]. There is no UFSM provision beyond this age group. In September 2023, the Mayor of the London Borough of Tower Hamlets implemented a major policy change and extended UFSM provision to all secondary school pupils (aged 11-16y) in Tower Hamlets [3]. 

UFSM has several areas of potential impact. These include, improving child poverty, nutrition (e.g., reducing hunger, promoting healthier food choices), behaviour and attainment, and wider economic aspects [4]. A systematic review in 2021[5] also highlighted some positive effects of UFSM on uptake, diet, attendance, academic performance, body mass index (BMI) and school finance [5]. Out of the 47 studies included, only seven were UK-based and predominantly focused on the primary school population. Four of these studies considered UFSM [6–9] and three considered Universal Free School Breakfasts [10–12]. The outcomes for UFSM were uptake[6], diet[7, 8], and uptake and cost combined [9]. Two further UK studies have since explored the effect of UFSM on diet [13] and BMI [14]. Dietary findings emphasize the need for school food standards, along with the importance of food availability and choice [5, 7, 8, 13]. Uptake findings show increased participation [6, 9] and BMI findings show positive improvements [14]. Borbely et al. 2024 explored the effects of UFSM on school attendance and health outcomes in primary school-aged pupils, and found uptake increased but there was no evidence of an impact on attendance [15]. 

A systematic review in 2024[16], included six US-based studies and explored the effect of UFSM on meal participation, attendance, weight status and school suspensions. This review supports some of the positive effects of UFSM such as, increased school meal participation, and decreased obesity prevalence, however, no study reported on dietary intake or quality [16]. Research is needed to examine the nutritional impact of school meals[15], and the wider effects of UFSM on diet and food security [16]. 

Qualitative research on UFSM include a process evaluation [17] and parental views [18] in primary schools. Key challenges associated with UFSM provision included food quality and choice[18], and a lack of staff knowledge on UFSM benefits [17]. In secondary schools, a process evaluation [18] and mixed methods study [19] explored stakeholder views and quantitative data on food insecurity (FI) and hunger [19, 20]. The authors aimed to explore the impact of UFSM on students hunger, behaviour and food consumption, along with the effects on family finance and food security. The data collected captured findings from a student survey, interviews with students, parents and school staff and student observations at lunchtime. Although stakeholders discussed positive impacts on FI and hunger, the quantitative data did not find that UFSM had a significant effect on FI and hunger [20]. 

One key premise of UFSM is that by offering a UFSM, dietary intakes will improve. Whilst children consuming school lunches have an improved nutritional intake compared with home-packed lunches, it is important to emphasize that evidence for this largely comes from primary school-aged children [21, 22]. Less evidence exists to support this in secondary school-aged children [23, 24]. The implementation of UFSM in Tower Hamlets is a major policy change and, whilst this has potential for beneficial impacts on secondary school-aged pupils’ diets and food security, few opportunities exist to explore these effects. Tower Hamlets committed to providing all secondary school-aged pupils a free school meal. Pupils who would normally pay for a school meal were also eligible for a free school meal. Schools continued to have a variety of food and drink options on their weekly menus. Given there is limited evidence about the effects of UFSM in secondary school-aged children, this research is of particular relevance to wider discussions on universal provision of school food. This change in UFSM provision offered an opportune natural experiment to explore the effects of UFSM on secondary-school aged pupils’ dietary intake and food security.

## Methods

### Aim

To explore the effects of UFSM on secondary-school aged pupils’ dietary intake and food security.

### Study design and setting

We undertook a natural experiment using a mixed longitudinal cross-sectional study design at two timepoints: pre- (June/July & Sept 2023) and post-UFSM implementation (October 2023 - June 2024) in three secondary schools in Tower Hamlets considered as ‘intervention’ schools. Data were also collected at two time-points (January - June 2024) from control schools in a London Borough where UFSM was not implemented. Although three control schools were recruited, only two completed data collection. The comparator Borough cannot be disclosed for confidentiality reasons. It was selected through discussion with the Tower Hamlets Public Health Team who liaised with other London Boroughs to identify a Borough with a similar population. Schools in the comparator borough were considered based on percentage Free School Meal (FSM) eligibility and school type (i.e., non-Academy).

### Recruitment: schools and pupils

All schools were recruited by liaising with Public Health contacts in the respective London Boroughs. Public Health contacts disseminated an email with the project information to all secondary school heads. School heads contacted the research team and Zoom meetings were arranged to explain the study and answer questions.

Schools purposefully selected two or three Year 8 classes to provide approximately 60 pupils per school. All pupils within these classes were invited to participate. Recruitment was restricted to Year 8 pupils to avoid interference with school examinations in older age groups. All schools were given the option for the researchers to join Year 8 classes via Zoom to talk about the study and answer questions. Parental/guardian study information letters and opt-out consent forms were circulated by schools through their communication platforms using email or texts. Parent/guardians completed the consent form if they did not want their child to participate via a Microsoft Office Forms link which was returned directly to researchers. Pupil names and postcodes were provided via a secure File Drop-Off service. Schools received a voucher, and pupils received a £20 voucher on completion.

### Data collection

Data collection was completed during an agreed timetabled session. Prior to this, each school contact received a word document via a secure File Drop-Off that contained participating pupils’ names and two unique survey links beside each individual pupil name. Survey one contained a unique Intake24 URL that each individual pupil logged on to and completed their 24-hour dietary recall [25]. Survey two contained a unique Qualtrics [26] link for the pupil to complete the child food security questions. Each school received a password protected Zoom link for researchers to join and answer pupil questions through the school contact using the chat function or verbal communication.

### Dietary

Pupils completed four 24-hour dietary recalls on non-consecutive weekdays using Intake24. In intervention schools, pupils recorded dietary data for two days pre- and for two days in the post-UFSM periods. In control schools, pupils recorded two days of dietary intake on two separate occasions. Intake24 is an online multiple pass 24-hour dietary recall tool previously validated in this age group [27]. Pupils recalled all food and drink consumed during the prior 24-hour period. If a pupil could not find their food, they selected ‘I can’t find my food’ and tried different search terms. Failing this, they selected ‘report a missing food’ and answered questions to provide relevant information. Pupils estimated portion size using photographs in Intake24, recorded ‘food source’ (e.g. school, shop) and meal type (e.g. breakfast, lunch). Pupils were allocated a lunch-type based on food source.

Food and drink within Intake24 are automatically linked to the NDNS Nutrient Databank [28] and food groups. Dietary data were exported from Intake24 into Microsoft Excel spreadsheet. Intake24 automatically flags ‘unreasonable’ portion sizes, any foods flagged were discussed and an agreed average portion size based on published age-appropriate average portion sizes was allocated [29]. Fruit and vegetable portions were calculated following the NDNS method [30]. Dietary data were checked for under (< 400 kcal) and over-reporting (> 4000 kcal) following the NDNS protocol.

### Food security

The child food security questionnaire (survey two) was completed once pre- and post-UFSM for pupils in intervention schools and in control schools. The Child Food Security Survey Module measured food insecurity [31]. This includes nine questions for the prior 30-day reference period (e.g., ‘*did you worry that food at home would run out before your family got money to buy more*?’ Additional File 1). Each question had three responses: a lot, sometimes, never. The sum of scores for each question were combined and categorised (Table 1).

**Table 1:** Classification of child food security based on the Raw scores from the nine questions

Raw scores	Food Security	Categorisation
0	High	Food Secure
1	Marginal	Food Secure
2–5	Low	Food Insecure
6–9	Very low	Food Insecure

### Demographics

Information on pupil postcodes and gender were collected. Postcodes were mapped to the English Index of Multiple Deprivation (IMD) 2019 scores, and categorised into five quintiles using national cut-off points [32]. 

### Main outcome measures

Main dietary outcomes were mean change pre-post UFSM at lunchtime and in total diet in: Energy (kcal), per cent energy non-milk extrinsic sugars (NMES), saturated fat and fat, protein (g), fibre (AOAC) (g), sodium (mg), calcium (mg), iron (mg) and fruit & vegetables (portions).

### Statistical analysis

#### Structure

The first analysis provides a description of pupil gender and IMD status. The aim of further analyses is to compare the intervention and control schools with respect to pertinent pupil choices and important nutritional variables. As intervention and control schools could not be chosen randomly, some account needed to be taken of potential differences between schools in the two groups. This has been done by focussing the analysis on within-pupil changes between periods that corresponded to pre- to post-UFSM in the intervention schools, i.e., using paired data from pupils with data in both periods. Data on changes in lunch-types and food security are tabulated.

#### Modelling

Within-pupil changes in nutritional variables were analysed using a linear model that allowed for difference due to gender, intervention or control school and lunch-type. Lunch-type was categorised as: School, for those having school lunch (SL – this includes paid and FSM pupils) in both periods; Home, for those having a home-prepared lunch in both periods; Switched to SL for those changing to a school lunch between the periods of the study and Switched from SL for those changing from a school lunch; all other transitions are classified as Other. As nothing systematic changed between the periods for those not changing lunch-types, a simple main effect for intervention/control would be difficult to interpret, so a lunch-type by intervention/control interaction was fitted.

#### Reporting

The *p*-values for the lunch-type by intervention/control interactions from the analyses of variance of the fitted models are reported. Mean changes (adjusted for gender) for lunch-type by intervention and control are tabulated. Differences in the adjusted mean changes between intervention and control, and associated 95% confidence intervals, are also presented in these tables to assess the effect of the introduction of UFSM. All analyses were conducted in Stata V18.

## Results

### Participant characteristics

Table 2 shows the number of pupils by intervention/control school, gender and level of deprivation. Although 236 pupils had some dietary data, the analyses were restricted to the 176 pupils with data in both periods, 99 from the three intervention schools and 77 from the two control schools. There was little difference in key characteristics (e.g. gender, IMD, lunch-type) of pupils included and not included. In the sample of 176 pupils, there were more females than males, due to an all-girl school in both the intervention and control groups. The majority of pupils were in the two most deprived IMD quintiles (IMD quintile 1 and 2) regardless of school type. In total, 93% (*n* = 92) and 98% (*n* = 75) of pupils are in the two most deprived IMD quintiles in the intervention and control schools respectively, although there is some difference in how this is distributed (Table 2).

**Table 2:** Sample characteristics by intervention and control schools

	Intervention Schools (*n* = 3) *n*(%)	Control Schools (*n* = 2) *n*(%)
Gender		
Female	63 (64)	61 (79)
Male	36 (36)	16 (21)
Level of deprivation (Quintiles)		
1 (most deprived)	27 (27)	39 (51)
2	65 (66)	36 (47)
3	6 (6)	2 (2)
4	0 (0)	0 (0)
5 (least deprived)	1 (1)	0 (0)
Total	99 (100)	77 (100)

### Change in pupils’ lunch-type pre-post UFSM implementation and child food security scores

Table 3 shows the actual number and percentage of pupils’ lunch-type pre- and post-UFSM, and by intervention or control school. It highlights similar percentages of pupils having a school lunch pre-UFSM in intervention and control schools (36% and 39% respectively) and post- UFSM (36% and 40%). This is similar for home lunches.

**Table 3:** Actual number (%) of pupils’ lunch-type pre- and post-UFSM, and by intervention or control schools

	Intervention (*n* = 99)				Control (*n* = 77)			
	School	Home	Other	Unknown	School	Home	Other	Unknown
Pre *n*(%)	36 (36)	38 (38)	14 (14)	11 (11)	30 (39)	30 (39)	12 (16)	5 (6)
Post *n(%)*	36 (36)	29 (29)	29 (29)	5 (5)	31 (40)	23 (30)	12 (16)	11 (14)

The changes in pupil lunch-types pre- and post-UFSM, by school type are shown in Table 4. There are several different lunch-type combinations, e.g. the numbers of pupils consuming a school lunch both pre- and post-UFSM in intervention and control schools is 17 and 19 respectively. Only a small number of pupils switched from a home, other and unknown to a school lunch post-UFSM in intervention and control schools (19 and 12 respectively).

**Table 4:** The change in pupil lunch-type pre- and post-UFSM, and by intervention or control schools

		Intervention (*n* = 99)					Control (*n* = 77)			
		Post					Post			
		School	Home	Other	Unknown		School	Home	Other	Unknown
Pre	School	17	5	11	3	School	19	3	3	5
	Home	9	16	12	1	Home	6	15	6	3
	Other	3	4	6	1	Other	4	5	2	1
	Unknown	7	4	0	0	Unknown	2	0	1	2

Complete pupil food security data was available from 149 pupils. Most pupils were food secure both pre- and post-UFSM in intervention and control schools (67% and 53% respectively) (Table 5). In intervention and control schools the change from food secure to food insecure, or vice versa was similar. In intervention schools, there was a lower percentage of pupils that were food insecure both pre- and post-UFSM in comparison to the control schools (14% v 29% respectively).

**Table 5:** Change in number (%) of pupils’ food security pre- and post-UFSM and by school type

		Intervention (*n* = 79)		Control (*n* = 70)	
		Post *n*(%)		Post *n*(%)	
		Food secure	Food Insecure	Food secure	Food insecure
Pre	Food secure	53 (67)	5 (6)	37 (53)	5 (7)
	Food insecure	10 (13)	11 (14)	8 (11)	20 (29)

### Dietary intake

#### Total diet: the effect of a lunch-type and intervention/control interaction on mean change in pupils macro- and micro-nutrient intakes

From the analyses of variance of the fitted models we found no evidence of an effect of a lunch-type and intervention/control on mean change in pupils’ intake of energy (kcals) (*p* = 0.36), per cent energy from non-milk extrinsic sugars (NMES) (*p* = 0.84), saturated fat (*p* = 0.39) or fat (*p* = 0.62). There was also no evidence of lunch-type and intervention/control interaction on mean change in pupil’s intake of protein (g) (*p* = 0.14), fibre (g) (*p* = 0.32), sodium (mg) (*p* = 0.07), iron (*p* = 0.43) or fruit and vegetable portions (*p* = 0.10).

The mean changes for each lunch-type by intervention and control schools are shown in Table 6 for energy intake and per cent energy from NMES, saturated fat and fat, together with the difference in these mean changes and its associated 95% confidence interval. An analogous table for the intakes of protein, fibre, sodium, iron and fruit & vegetables is presented in Additional File 2.

**Table 6:** The effect of a lunch-type and intervention/control interaction on pupils' mean nutrient intakes (Total Diet)

		School (*n* = 176)				
		Intervention (*n* = 99)	Control (*n* = 77)			
Nutrient	Lunch-type	mean change (post - pre UFSM)*		Difference in changes^†^	95% CI^ǂ^	
Energy (kcals) ^§^	School Lunch	−164.6	−29.2	−135.4	−569.8	299.1
	Home Packed	31.1	−40.7	71.7	−394.8	538.3
	Switched from SL	−98.7	−35.2	−63.5	−560.7	433.7
	Switched to SL	185.5	−311.6	497.1	17.3	976.9
	Other	38.9	33.5	5.4	−376.3	387.2
%E NMES ^¶^	School Lunch	1.6	1.7	0.1	−6.7	6.5
	Home Packed	3.3	−2.2	5.5	−1.6	12.6
	Switched from SL	−1.4	−2.9	1.5	−6.1	9.0
	Switched to SL	3.9	0.5	3.4	−3.9	10.7
	Other	2.7	0.5	2.2	−3.6	8.1
%E Saturated Fat	School Lunch	−2.2	0.6	−2.8	−6.2	0.7
	Home Packed	−3.1	0.7	−3.8	−7.5	−0.1
	Switched from SL	−0.5	0.9	−1.4	−5.4	2.5
	Switched to SL	−1.6	0.9	−2.6	−6.4	1.2
	Other	1.9	1.3	0.6	−2.4	3.7
%E Fat	School Lunch	1.2	0.9	0.3	−6.1	6.7
	Home Packed	−2.1	3.2	−5.3	−12.1	1.6
	Switched from SL	−3.3	−0.3	−3.0	−10.3	4.4
	Switched to SL	0.0	0.7	−0.7	−7.7	6.4
	Other	1.8	0.4	1.4	−4.3	7.0

*adjusted for gender, *UFSM *Universal Free School Meals

^†^(mean change intervention (post-pre UFSM) - mean change control (post-pre UFSM))

^ǂ^ 95% Confidence Interval

^§^ kilocalories

^¶^ per cent energy non-milk extrinsic sugars

#### Lunchtime: the effect of a lunch-type and intervention/control interaction on mean change in pupils macro- and micro-nutrient intakes

Similar to total diet, we found no evidence of lunch-type and intervention/control interaction on mean change for most variables (*p*-values from analysis of variance): pupils’ intake of per cent energy from non-milk extrinsic sugars (NMES) (*p* = 0.82), saturated fat (*p* = 0.23) or fat (*p* = 0.43), fibre (g) (*p* = 0.14), sodium (mg) (*p* = 0.10), iron (mg) (*p* = 0.24) or fruit and vegetable portions (*p* = 0.69). There was some evidence of an interaction for energy intake (kcals) (*p* = 0.02) and intake of protein (*p* = 0.03).

Following the presentation for total diet, the mean changes for each lunch-type by intervention and control schools for energy and per cent energy variables are shown in Table 7. An analogous table for the intakes of protein, fibre, sodium, iron and fruit & vegetables is presented in Additional File 3. From Table 7, the significant result for energy intake seems to be because pupils in the intervention schools in the Other category now consume a higher mean energy intake (115.1 kcals); pupils in control schools now consume a lower mean energy intake (−301.1 kcals) (mean difference 416·2 kcals; 95% CI 174.5; 657.9). From Additional File 3 the results for protein intake seems to be because pupils in the intervention schools that switched from a school lunch now consume a lower mean protein intake (−2.9 g); pupils in control schools that switched from a school lunch now consume a higher mean intake (18.6 g) (mean difference − 21.5 g; 95% CI −38.8; −4.3).

**Table 7:** The effect of a lunch-type and intervention/control interaction on pupils' mean nutrient intakes (lunchtime)

		School (*n* = 145)				
		Intervention	Control			
Nutrient	Lunch-type	mean change (post - pre UFSM)*		Difference in changes^†^	95% CI^ǂ^	
Energy (kcals) ^§^	School Lunch	−44.0	−54.8	10.8	−219.4	240.9
	Home Packed	20.9	−36.5	57.4	−189.7	304.4
	Switched from SL	1.2	247.1	−245.9	−581.2	89.4
	Switched to SL	−23.3	−187.0	163.7	−131.1	458.5
	Other	115.1	−301.1	416.2	174.5	657.9
%E NMES ^¶^	School Lunch	5.0	0.6	4.4	−5.8	14.6
	Home Packed	0.3	−0.8	1.1	−9.9	12.0
	Switched from SL	1.7	5.4	−3.7	−18.7	11.2
	Switched to SL	−0.1	4.6	−4.7	−17.9	8.3
	Other	3.9	4.6	−0.7	−11.5	10.1
%E Saturated Fat	School Lunch	1.6	−2.1	3.7	−7.1	14.5
	Home Packed	−2.8	−3.9	1.1	−10.4	12.8
	Switched from SL	1.0	−7.6	8.6	−7.2	24.3
	Switched to SL	−10.8	3.0	−13.8	−27.6	0.0
	Other	−2.7	−1.2	−1.5	−12.8	9.8
%E Fat	School Lunch	−4.4	1.5	−5.9	−17.5	5.6
	Home Packed	−2.3	−0.1	−2.2	−14.6	10.2
	Switched from SL	−2.3	6.7	−9.0	−25.8	7.9
	Switched to SL	4.1	3.9	0.2	−14.7	14.9
	Other	2.9	−5.0	7.9	−4.2	20.1

*adjusted for gender, *UFSM* Universal Free School Meals

^†^(mean change intervention (post-pre UFSM) - mean change control (post-pre UFSM))

^ǂ^ 95% Confidence Interval

^§^ kilocalories

^¶^ per cent energy non-milk extrinsic sugars

## Discussion

### Summary of key findings

A key finding was only a small number of pupils in the intervention schools changed lunch-type to take up the UFSM offer. Most pupils were found to have a combination of School, Home-packed or Other lunch across a school week. This was similar in control schools. Inspection of Table 4 reveals that over the study period many pupils change lunch-type (61% intervention; 51% control), indicating the difficulty of detecting any changes related to the intervention.

Only two of the 18 tests for interaction gave *p* < 0.05, namely for energy and protein intake in lunchtime diet. The source of these interactions, namely those in the Other category (energy) and those moving from a school lunch (protein), are hard to interpret in the context of the results as a whole, where most observed differences can be ascribed to chance. Given the number of hypothesis tests conducted, it is quite possible that these two effects are also due to chance. That no tests are significant is plausible given the small number of pupils that switched to take the UFSM offer.

### Relationship to other studies

Prior to the UFSM policy, uptake of FSM in Tower Hamlets’ secondary schools was low. Although 51% of pupils are eligible, only half of pupils eligible took up the offer [33]. This study found only a small number of pupils changed to a school lunch pre- to post-UFSM. Several factors such as the dining environment[34], and pupils from more deprived communities having a negative perception of school food[35], have been reported as impacting school lunch uptake. These may partially contribute to a poor uptake of UFSM in this study, however, some caution is required as there are no supporting qualitative findings.

In secondary schools a range of food and drink options are available daily - this may explain some variation in nutritional intake depending on pupil food choices. The lack of nutritional benefits is similar to other school-based studies involving secondary school-aged pupils. Pallan et al. 2024[36] found few statistically significant results when they explored the impact of school food standards on the dietary intakes of secondary school-aged pupils. They found no school achieved 100% compliance and schools were less compliant with standards that restricted high fat, sugar and energy dense food and drinks. To improve pupils’ nutritional intakes, the nutritional quality of foods and drink on offer, along with improving pupils’ food and drink choices in school, is required[24, 37, 38].

### Strengths and limitations

This is the first UK study to evaluate the effect of UFSM on secondary school-aged pupils’ actual dietary intake. Unique to this study is that dietary data were collected pre- and post-UFSM implementation and included pupils attending intervention and control schools. Previous timelines of UFSM implementation have not enabled a pre- to post- implementation evaluation in the same pupils, or comparison of intervention and control schools. In addition, potential dietary effects across the whole day (total diet) were explored, along with lunchtime effects.

There are several limitations. The number of schools and sample size of pupils was restricted by the timescale of the UFSM implementation to enable pre-policy data collection, nor was a randomized design possible. The sample may not be representative of the total population; pupils in both intervention and control schools were mainly from more deprived households. We cannot distinguish between a paid and FSM pupil. Although the policy was implemented in all intervention schools there is potential for school-level variation in UFSM implementation, and, food and/or drink options may have differed. Data collection in intervention schools was completed in the first few months of UFSM implementation. There may have been a delay between implementation and pupils taking up the offer. In addition, we do not know how this offer of UFSM was communicated to parents and/or pupils. Data collection in the control schools commenced after intervention schools, this was due to practical reasons, however, time trends were not anticipated. No qualitative research was included to explore pupil’s reasons for not taking up UFSM or gain insight from school staff about the implementation process.

Two days of dietary data were collected at two time-points, but not all pupils completed four days. Pupils were included in the analysis if they completed one or two days at both time-points. Some pupils (25%) were not included in these analysis as they had not completed dietary data collection at both time points. Although Intake24 is validated for use in this age group, we cannot remove the risk of pupil’s mis-reporting dietary intake [39]. In addition, the number of days that dietary data could be collected was limited to avoid overburdening schools and pupils. Food and drink on offer in schools varies daily, we did not collect school level detail as to the offer. These issues may limit identifying dietary effects.

## Conclusions and future research

This study has provided insight into several nuances associated with UFSM provision in secondary school-aged pupils. Although we found almost no evidence of an effect of the intervention on pupils’ dietary intakes, we must avoid implying UFSM does not affect secondary school-aged pupils’ dietary intakes. UFSM may have wider effects that we did not capture. A key reason for this is the important finding that only a small number of pupils in the intervention schools took up the UFSM offer within the time-period of this study.

In secondary schools, pupils have multiple daily combinations of lunch-type: school, home-packed, or mixed lunches. Implementing an approach where pupils take a school-lunch for a full week could be considered to maximise nutritional benefits for secondary school-aged pupils, and, from a school perspective be preferable financially. However, acceptability needs considered to avoid unintended consequences. Future research requires a mixed methods design to understand reasons for not taking up the UFSM offer. Follow-up studies may reveal if take-up increases over time. If UFSM take-up does increase, larger studies would be required to explore the effects on pupils who take up the offer.

## Supplementary Information

Additional file 1: Child Food Security Survey.

Additional file 2: The effect of a lunch-type and intervention/control interaction on pupils' mean nutrient intakes of protein (g), fibre (g), sodium (mg), iron (mg) and FV (portions) (Total Diet).

Additional file 3: The effect of a lunch-type and intervention/control interaction on pupils' mean nutrient intakes of protein (g), fibre (g), sodium (mg), iron (mg) and FV (portions) (Lunchtime).

## Data Availability

The dataset used during the current study are available from the corresponding author on reasonable request.

## Abbreviations

AOAC: Fibre
BMI: Body mass index
CI: Confidence interval
FI: Food insecurity
FSM: Free School Meals
g: Gram
IMD: Index of Multiple Deprivation
Kcal: Kilocalorie
mg: Milligram
N: Number
NDNS: National Diet and Nutrition Survey
NMES: Non-milk extrinsic sugars
PL: Packed lunch
SL: School lunch
UFSM: Universal Free School Meals
UK: United Kingdom
US: United States
