# Supplementary material for: An evaluation of the effects of universal free school meals on secondary school-aged pupils’ dietary intakes in England: a natural experiment
Source: BMC Public Health. 2025 Dec 19;26:299. doi: 10.1186/s12889-025-25960-7 (PMC12831372; doi:10.1186/s12889-025-25960-7)
Supplement: Supplementary file 1 — Additional file 1: Child Food Security Survey. [file 12889_2025_25960_MOESM1_ESM.pdf]

## Self-Administered Food Security Survey Module for Children Ages 12 Years and Older

September 2006

**Background:** The Child Food Security Survey Module was developed by researchers at the University of Southern Mississippi in collaboration with ERS and documented in "Food Security of Older Children Can Be Assessed Using a Standardized Survey Instrument," by Carol L. Connell, Mark Nord, Kristi L. Lofton, and Kathy Yadrick (published by the *Journal of Nutrition*, vol. 134, no. 10, pp. 2566-72, 2004). Internal validity of the module was found adequate for children ages 12 and older, but its use is not recommended for younger children.

Initial validation was conducted in a sample of children from a school in Mississippi. Use of the module in other regions of the country should be considered exploratory until further validation assessment is conducted.

Cognitive testing indicated that recall and responses for a 12-month period might not be reliable, and a 30-day reference period is recommended.

**[Begin Child Food Security Survey Module]**

The following questions are about the food situation in your home **during the last month**. Please circle the answer that best describes you. Do not put your name on the paper. Your answers will remain a secret.

1. Did you **worry** that food at home would run out before your family got money to buy more?
   _______ A LOT
   _______ SOMETIMES
   _______ NEVER

2. Did the food that your family bought **run out**, and you didn't have money to get more?
   _____ A LOT
   _____ SOMETIMES
   _____ NEVER

3. Did your meals only include a few kinds of **cheap foods** because your family was running out of money to buy food?
   _____ A LOT
   _____ SOMETIMES
   _____ NEVER

4. How often were you not able to eat a **balanced meal** because your family didn't have enough money?
   _____ A LOT
   _____ SOMETIMES
   _____ NEVER

5. Did you have to **eat less** because your family didn't have enough money to buy food?
   _____ A LOT
   _____ SOMETIMES
   _____ NEVER

6. Has the size of your meals **been cut** because your family didn't have enough money for food?
   _____ A LOT
   _____ SOMETIMES
   _____ NEVER

7. Did you have to **skip a meal** because your family didn't have enough money for food?
   _____ A LOT
   _____ SOMETIMES
   _____ NEVER

8. Were you **hungry** but didn't eat because your family didn't have enough food?
   _____ A LOT
   _____ SOMETIMES
   _____ NEVER

9. Did you not eat for a **whole day** because your family didn't have enough money for food?
   _____ A LOT
   _____ SOMETIMES
   _____ NEVER

**[End of Child Food Security Survey Module]**

## User Notes

**(1) Coding Responses and Assessing Children's Food Security Status:**
Responses of "a lot" or "sometimes" are coded as affirmative. The sum of affirmative responses to the nine questions in the Child Food Security Module is the respondent's raw score on the scale.

Provisional classification guidance (updated to be consistent with USDA's 2006 labels for other scales) is as follows:

- Raw score 0—High food security
- Raw score 1—Marginal food security
- Raw score 2-5—Low food security
- Raw score 6-9—Very low food security

For some reporting purposes, the food security status of youth with raw score 0-1 is described as food secure and the two categories "low food security" and "very low food security" in combination are referred to as food insecure.

For statistical procedures that require an interval-level measure, the following scale scores, based on the Rasch measurement model may be used:

| Number of affirmatives | Scale Score |
|---|---|
| 0 | NA |
| 1 | -0.1 |
| 2 | 1.9 |
| 3 | 3.4 |
| 4 | 4.7 |
| 5 | 5.9 |
| 6 | 7.2 |
| 7 | 8.7 |
| 8 | 10.8 |
| 9<br>(evaluated at 8.5) | 12.5 |

However, no interval-level score is defined for youth who affirm no items. (They are food secure, but the extent to which their food security differs from those who affirm one item is not known.)
