# Supplementary material for: An evaluation of the effects of universal free school meals on secondary school-aged pupils’ dietary intakes in England: a natural experiment
Source: BMC Public Health. 2025 Dec 19;26:299. doi: 10.1186/s12889-025-25960-7 (PMC12831372; doi:10.1186/s12889-025-25960-7)
Supplement: Supplementary file 2 — Additional file 2: The effect of a lunch-type and intervention/control interaction on pupils' mean nutrient intakes of protein (g), fibre (g), sodium (mg), iron (mg) and FV (portions) (Total Diet). [file 12889_2025_25960_MOESM2_ESM.docx]

**Additional File 2** The effect of a lunch-type and intervention/control interaction on pupils mean nutrient intakes of protein (g), fibre (g), sodium (mg), iron (mg) and FV (portions) (Total Diet)

|  |  | **School Type (n=176)** | |  |  |  |
| --- | --- | --- | --- | --- | --- | --- |
|  |  | **Intervention** | **Control** |  |  |  |
| **Nutrient** | **Lunch-type** | **mean change**  **(post – pre UFSM)*** | | **Difference in changes**^†^ | **95% CI****^ǂ^** | |
| **Protein (g)**^§^ | School Lunch | -13.1 | -9.9 | -3.2 | -25.6 | 19.3 |
|  | Home Packed | -9.7 | 6.3 | -16.0 | -40.1 | 8.1 |
|  | Switched from SL | -5.8 | -2.2 | -3.6 | -29.3 | 22.0 |
|  | Switched to SL | 16.4 | -11.4 | 27.8 | 3.1 | 52.6 |
|  | Other | 2.2 | -3.1 | 5.3 | -14.3 | 25.0 |
| **Fibre (g)** | School Lunch | -1.6 | 0.8 | -2.4 | -6.9 | 2.1 |
|  | Home Packed | 1.5 | 0.7 | 0.8 | -4.1 | 5.6 |
|  | Switched from SL | -0.1 | 0.2 | -0.3 | -5.4 | 4.9 |
|  | Switched to SL | 0.6 | -2.4 | 3.0 | -2.0 | 7.9 |
|  | Other | -2.6 | 0.6 | -3.2 | -7.2 | 0.7 |
| **Sodium (mg)**^¶^ | School Lunch | -444.2 | -21.4 | -422.8 | -1139.8 | 294.2 |
|  | Home Packed | -123.1 | 107.9 | -231.0 | -1001.1 | 539.0 |
|  | Switched from SL | -246.2 | -25.4 | -220.8 | -1041.4 | 599.8 |
|  | Switched to SL | 515.2 | -517.5 | 1032.7 | 240.8 | 1824.6 |
|  | Other | 22.6 | -81.5 | 104.1 | -526.0 | 734.3 |
| **Iron (mg)** | School Lunch | -5.7 | -1.4 | -4.3 | -9.7 | 1.1 |
|  | Home Packed | -0.6 | 0.3 | -0.9 | -6.7 | 4.9 |
|  | Switched from SL | 2.9 | 1.0 | 1.9 | -4.4 | 8.0 |
|  | Switched to SL | 1.6 | -0.9 | 2.5 | -3.4 | 8.5 |
|  | Other | -0.5 | -1.6 | 1.1 | -3.7 | 5.8 |
| **FV (portions)** | School Lunch | 0.0 | 0.2 | -0.2 | -1.4 | 1.0 |
|  | Home Packed | 1.2 | -0.7 | -1.9 | -0.6 | -3.2 |
|  | Switched from SL | -0.1 | 0.4 | 0.5 | -1.9 | 0.9 |
|  | Switched to SL | -0.5 | -0.5 | 0.0 | -1.3 | 1.4 |
|  | Other | 0.2 | -0.1 | -0.3 | -0.7 | 1.4 |

*adjusted for gender; Universal Free School Meals (UFSM); ^†^(mean change intervention (post-pre UFSM) - mean change control (post-pre UFSM)); **^ǂ^** 95% Confidence Interval; ^§^ grams; ^¶^ milligrams
