# Supplementary material for: An evaluation of the effects of universal free school meals on secondary school-aged pupils’ dietary intakes in England: a natural experiment
Source: BMC Public Health. 2025 Dec 19;26:299. doi: 10.1186/s12889-025-25960-7 (PMC12831372; doi:10.1186/s12889-025-25960-7)
Supplement: Supplementary file 3 — Additional file 3: The effect of a lunch-type and intervention/control interaction on pupils' mean nutrient intakes of protein (g), fibre (g), sodium (mg), iron (mg) and FV (portions) (Lunchtime). [file 12889_2025_25960_MOESM3_ESM.docx]

**Additional File 3** The effect of a lunch-type and intervention/control interaction on pupils mean nutrient intakes of protein (g), fibre (g), sodium (mg), iron (mg) and FV (portions) (Lunchtime)

|  |  | **School Type (*n*=145)** | |  |  |  |
| --- | --- | --- | --- | --- | --- | --- |
|  |  | **Intervention** | **Control** |  |  |  |
| **Nutrient** | **Lunch-type** | **mean change**  **(post - pre UFSM)*** | | **Difference in changes**^†^ |  | **95% CI^ǂ^** |
| **Protein (g)** ^§^ | School Lunch | -2.5 | -3.0 | 0.5 | -11.3 | 12.3 |
|  | Home Packed | 0.6 | -0.9 | 1.5 | -11.2 | 14.2 |
|  | Switched from SL | -2.9 | 18.6 | -21.5 | -38.8 | -4.4 |
|  | Switched to SL | 5.4 | -5.4 | 10.8 | -4.3 | 25.9 |
|  | Other | 2.1 | -9.6 | -11.7 | -0.7 | 24.1 |
| **Fibre (g)** | School Lunch | -0.1 | -0.4 | 0.3 | -2.1 | 2.7 |
|  | Home Packed | 0.2 | 0.3 | -0.1 | -2.6 | 2.5 |
|  | Switched from SL | 0.6 | 1.2 | -0.6 | -4.1 | 2.9 |
|  | Switched to SL | -1.0 | -1.7 | 0.7 | -2.4 | 3.8 |
|  | Other | 0.9 | -2.9 | 3.8 | 1.4 | 6.4 |
| **Sodium (mg)** ^¶^ | School Lunch | -181.4 | -79.8 | -101.6 | -519.1 | 316.0 |
|  | Home Packed | -9.7 | -3.9 | -5.8 | -454.0 | 442.4 |
|  | Switched from SL | -130.9 | 147.6 | -278.5 | -886.8 | 329.3 |
|  | Switched to SL | 53.3 | -178.2 | 231.5 | -303.3 | 766.4 |
|  | Other | 110.7 | -478.5 | 589.2 | 150.7 | 1027.7 |
| **Iron (mg)** | School Lunch | -0.2 | 0.5 | -0.7 | -11.5 | 10.1 |
|  | Home Packed | 0.5 | 0.4 | 0.1 | -11.4 | 11.7 |
|  | Switched from SL | -1.5 | 1.7 | -3.2 | -18.9 | 12.4 |
|  | Switched to SL | 16.7 | -0.4 | 17.1 | 3.3 | 30.9 |
|  | Other | -0.4 | -1.3 | 0.9 | -10.4 | 12.2 |
| **FV (portions)** | School Lunch | 0.3 | 0.1 | 0.2 | -0.5 | 0.7 |
|  | Home Packed | 0.6 | 0.0 | 0.6 | 0.0 | 1.3 |
|  | Switched from SL | 0.1 | 0.2 | -0.1 | -1.0 | 0.8 |
|  | Switched to SL | -0.3 | -0.4 | 0.1 | -0.6 | 0.9 |
|  | Other | 0.2 | 0.1 | 0.1 | -0.6 | 0.7 |

*adjusted for gender; Universal Free School Meals (UFSM); ^†^(mean change intervention (post-pre UFSM) - mean change control (post-pre UFSM)); **^ǂ^** 95% Confidence Interval; ^§^ grams; ^¶^ milligrams
